# Evaluation of renal sodium handling in heart failure with preserved ejection fraction: A pilot study

**DOI:** 10.14814/phy2.16033

**Published:** 2024-05-13

**Authors:** Adhish Agarwal, Srinivasan Beddhu, Robert Boucher, Veena Rao, Nirupama Ramkumar, Aylin R. Rodan, Jacob Fang, Brandi M. Wynne, Stavros G. Drakos, Thomas Hanff, Alfred K. Cheung, James C. Fang

**Affiliations:** ^1^ Division of Nephrology and Hypertension, Department of Internal Medicine University of Utah Salt Lake City Utah USA; ^2^ Medical Service, Veterans Affairs Salt Lake City Health Care System Salt Lake City Utah USA; ^3^ Yale University School of Medicine New Haven Connecticut USA; ^4^ Department of Human Genetics University of Utah Salt Lake City Utah USA; ^5^ Division of Cardiovascular Medicine, Department of Internal Medicine University of Utah Salt Lake City Utah USA

**Keywords:** heart failure with preserved ejection fraction, renal sodium handling

## Abstract

The pathophysiology behind sodium retention in heart failure with preserved ejection fraction (HFpEF) remains poorly understood. We hypothesized that patients with HFpEF have impaired natriuresis and diuresis in response to volume expansion and diuretic challenge, which is associated with renal hypo‐responsiveness to endogenous natriuretic peptides. Nine HFpEF patients and five controls received saline infusion (0.25 mL/kg/min for 60 min) followed by intravenous furosemide (20 mg or home dose) 2 h after the infusion. Blood and urine samples were collected at baseline, 2 h after saline infusion, and 2 h after furosemide administration; urinary volumes were recorded. The urinary cyclic guanosine monophosphate (ucGMP)/plasma B‐type NP (BNP) ratio was calculated as a measure of renal response to endogenous BNP. Wilcoxon rank‐sum test was used to compare the groups. Compared to controls, HFpEF patients had reduced urine output (2480 vs.3541 mL; *p* = 0.028), lower urinary sodium excretion over 2 h after saline infusion (the percentage of infused sodium excreted 12% vs. 47%; *p* = 0.003), and a lower baseline ucGMP/plasma BNP ratio (0.7 vs. 7.3 (pmol/mL)/(mg/dL)/(pg/mL); *p* = 0.014). Patients with HFpEF had impaired natriuretic response to intravenous saline and furosemide administration and lower baseline ucGMP/plasma BNP ratios indicating renal hypo‐responsiveness to NPs.

## 1 INTRODUCTION

Heart failure (HF), characterized by fluid overload, (Metra & Teerlink, 2017) affects over five million Americans and has a 5‐year survival of less than 50%. (Taylor et al., 2019) About half of all HF patients have a preserved cardiac ejection fraction (HFpEF), defined as >50%. (Owan et al., 2006) Most of the >1 million annual HF hospital admissions in the United States are due to HFpEF rather than HF with reduced EF (HFrEF).

Activation of the renin‐angiotensin‐aldosterone system (RAAS) and sympathetic nervous system are central to the pathophysiology of sodium and fluid retention in patients with HFrEF. (Braunwald, 2013) In contrast, little evidence confirms such neurohormonal activation as the cause for sodium and fluid retention in HFpEF. Although several different pathophysiological mechanisms likely explain the clinical heterogeneity of HFpEF, (Lam et al., 2009; Zamani et al., 2016) a distinct central pathophysiologic pathway that explains sodium and fluid avidity in HFpEF is sought. (Oghlakian et al., 2011; Tschope et al., 2017) Since sodium and fluid balance are primarily regulated by the kidney, defining alterations in the renal response to sodium loads and/or a diuretic challenge may clarify the mechanisms underlying congestion in HFpEF.

Natriuretic peptides (NPs) have diuretic, natriuretic, and vasodilating effects. (Brunner‐La Rocca & Sanders‐van, 2019) These physiological effects of NPs are largely mediated by the intracellular messenger, cyclic guanosine monophosphate (cGMP), and urinary cGMP levels reflect the renal response to NPs. (Butt et al., 2023; Wong et al., 1988) It has been postulated that an attenuated renal response to NPs, as evidenced by low urinary cGMP to plasma BNP ratio, contributes to the pathogenesis of sodium retention in HFrEF. (Jhund et al., 2017; Lourenco et al., 2009; Margulies et al., 1991) Furthermore, a decreased urinary cGMP to plasma BNP ratio portends a poor prognosis in HFrEF. (Butt et al., 2023; Lourenco et al., 2009) However, this relationship has not been fully explored in HFpEF.

We hypothesized that in a clinically euvolemic state, patients with HFpEF have impaired natriuretic and diuretic responses to sodium, volume and diuretic challenges, and have an attenuated renal response to endogenous BNP. To test this hypothesis, we conducted an open label, interventional pilot study in clinically euvolemic patients with HFpEF and control participants.

## 2 METHODS

This interventional, single‐center pilot study was conducted in the Clinical Research Unit of the Center for Clinical & Translational Science at the University of Utah (ClinicalTrials.gov ID NCT03837470). The study complies with the Declaration of Helsinki, and the University of Utahs institutional review board approved the study protocol. Written informed consent was obtained from each participant. The study included 14 participants of which nine were patients with HFpEF and five were control participants.

### 2.1 Study population

Patients with scheduled visits in the HF Clinic at the University of Utah were screened for eligibility with chart review. Potential participants were contacted, and informed written consent was obtained from those who chose to participate. Patients were eligible for study inclusion if they were adults aged 18–80 years, were able to give informed written consent, had a history of chronic (>6 months) HF with current New York Heart Association (NYHA) class I, II, or III symptoms, left ventricular ejection fraction (LVEF) >50% on a clinically indicated echocardiogram obtained within 12 months, and clinically compensated HF on stable medical therapy for HF without change in HF medications (including diuretics) for at least 7 days prior to the study. Exclusion criteria are detailed in the supplemental materials (10.6084/m9.figshare.24582234). Control volunteers were recruited through general announcements; included individuals aged 18–80 years with no prior history of cardiac or kidney disease, free of symptoms and a normal clinical cardiovascular examination on the day of study.

### 2.2 Study design and methods

After the study eligibility had been confirmed and an informed written consent obtained, the participants were scheduled for an 8 h study visit at the Clinical Research Unit. Demographic and clinical information were collected and recorded. In addition, home medications, including daily doses of diuretics were recorded. All patients followed a standardized protocol during the study visit, summarized in the Supplemental Figure (10.6084/m9.figshare.24582234).

Participants were instructed to consume a low (2–3 g/day) sodium diet for a week before the study and to hold their diuretics on the day of the study. On the day of the study, a physician investigator confirmed each participant's euvolemic status and clinical stability by performing a history and physical examination. Baseline blood and urine samples were collected. Participants were asked to void their bladder for baseline urine collection. Bladder ultrasonography was used to confirm adequate voiding (less than 100 mL of urine in their bladder) before 0.9% saline infusion at 0.25 mL/kg/min for 60 min was started. Blood and urine samples were collected 2 h after completion of the saline infusion. This was followed by a single intravenous bolus injection of furosemide (Hospira pharmaceuticals). The daily home oral furosemide dose was converted to a single intravenous dose at the ratio of 1:1, up to a maximum amount of 120 mg. If the participant was diuretic‐naïve or if the home dose was ≤20 mg daily, the intravenous furosemide dose was 20 mg. If the participant was on bumetanide at home, the bumetanide dose was converted to furosemide dose at a ratio of 1:40 (bumetanide: furosemide). None of the participants were on thiazide diuretics. Blood and urine samples were again collected 2 h after furosemide administration. Participants were observed for clinical stability for at least additional 15 min after the last sample collection before discharge.

All patients received a 2 g sodium diet during the study and had free access to water. Oral fluid intake and urine output were monitored and documented carefully. Blood pressures and heart rates were measured and recorded every 30 min, and the patient's heart rhythms were monitored with telemetry.

### 2.3 Laboratory measurements

Venous blood and urine samples were collected as detailed above. At baseline, blood samples were collected and sent to ARUP laboratories for measuring comprehensive metabolic panel, complete blood count, cystatin C, N‐terminal pro‐B‐type natriuretic peptide (NT‐pro BNP), BNP, plasma renin activity, aldosterone, epinephrine, norepinephrine, osmolality, and C‐reactive protein. Urine samples were tested for urinalysis, protein/creatinine ratio, diuretic screen, Na, K, Cl, creatinine, and osmolality (Freezing point method). Laboratory data were collected again 2 h after saline infusion ended (post‐infusion collection) and 2 h after furosemide injection (post‐furosemide collection).

### 2.4 Renal natriuretic response

Renal natriuretic response to saline infusion and intravenous furosemide was evaluated by calculating the percentage of the infused sodium that was excreted in the urine, and by measuring the fractional excretion of sodium (FeNa) (calculated using the formula [(urine Na × serum Cr)/(serum Na × urine Cr)]. (Alsaad & Wadei, 2016)). We also measured “diuretic efficiency” in the two groups by calculating the ratio of urinary sodium (in millimoles excreted over 2 h after furosemide injection) and furosemide dose administered (in milligrams).

### 2.5 Renal response to endogenous BNP

The renal response to endogenous BNP in the two groups was assessed by measuring the urinary cGMP to plasma BNP ratios. Urinary cGMP was measured from samples collected at baseline and after furosemide using competitive enzyme immunoassay (sensitivity of the assay was 3.06 pmol/mL and the assay range was 2.1–500 pmol/mL, R&D Systems Minneapolis, USA, Kit Catalog # KGE003) in a research laboratory at Yale University in New Haven, Connecticut. Measured urinary cGMP concentration was normalized for the urine creatinine concentration. Plasma BNP was measured from the blood samples as described previously.

### 2.6 Statistical analysis

Baseline and follow‐up characteristics of the participants were summarized in the HFpEF group and controls separately. Median and interquartile range (IQR) of variables in these two groups were displayed separately using box plots. We compared variables between the groups using one‐way analysis of variance (ANOVA) or Wilcoxon rank‐sum for continuous variables as appropriate, and chi‐square tests for categorical variables. All analyses were performed in STATA version MP 15.1.

## 3 RESULTS

Of the 14 participants in this pilot study, nine had HFpEF and five were healthy controls. HFpEF participants were older, had a higher baseline prevalence of diabetes mellitus, hypertension and coronary artery disease, higher body mass index (BMI), lower estimated glomerular filtration rate (eGFR) and higher systolic blood pressure as compared to controls (Table 1).

**TABLE 1:** Baseline characteristics of the study sample.

	All patients (*N* = 14)	Controls (*N* = 5)	HFpEF patients (*N* = 9)	*p*‐value
Age (years), mean (SD)	57 ± 16	47 ± 18	62 ± 12	0.075
Female, *n* (%)	8 (57)	3 (60)	5 (56)	0.870
Wt. (kg), mean (SD)	94 ± 34	71 ± 13	107 ± 36	0.057
BMI (kg/m^2^), mean (SD)	32.1 ± 9.1	24.6 ± 3.7	36.3 ± 8.5	0.014
Race, White (%)	100	100	100	N/A
Systolic blood pressure (mmHg), mean (SD)	125 ± 16	117 ± 9	129 ± 17	0.160
Heart rate (bpm), mean (SD)	68 ± 13	64 ± 14	69 ± 13	0.490
LVEF (%), mean (SD)			63 ± 5	N/A
Hypertension, *n* (%)	8 (57)	2 (40)	6 (67)	0.330
Diabetes Mellitus, *n* (%)	4 (29)	0 (0)	4 (44)	0.078
Coronary artery disease, n (%)	4 (29)	0 (0)	4 (44)	0.078
*NYHA, n*				N/A
I		N/A	1	
II		N/A	6	
III		N/A	2	
*Medication*				
Furosemide (loop diuretic), *n* (%)	5 (36)	0 (0)	5 (56)	0.038
ACEi or ARB, *n* (%)	4 (29)	1 (20)	3 (33)	0.600
Spironolactone, *n* (%)	5 (36)	0 (0)	5 (56)	0.038
*Laboratory parameters*				
Na (mmol/L), median (IQR)	138 (136, 141)	141 (139, 141)	136 (134, 138)	0.030
K, (mmol /L), median (IQR)	4.1 (3.9, 4.4)	3.9 (3.9, 4.1)	4.2 (4.0, 4.6)	0.140
Serum creatinine (mg/dL), median (IQR)	1.0 (0.9, 1.2)	0.8 (0.8, 0.9)	1.2 (1.0, 1.5)	0.009
CKD‐EPI (2021) eGFR (ml/min/1.73m^2^), mean (SD)	75 ± 26	100 ± 12	61 ± 21	0.003
FeNa (%), median (IQR)	0.005 (0.004, 0.008)	0.006 (0.004, 0.008)	0.004 (0.002, 0.008)	0.592
Serum aldosterone (ng/dL), median (IQR)	12 (6, 22)	6 (5, 6)	18 (14, 26)	0.003
BNP (pg/mL), median (IQR)	43.0 (5.0, 89.0)	5 (5, 34)	54 (29, 118)	0.150
NT‐proBNP (pg/mL), median (IQR)	129 (18, 200)	18 (15, 43)	197 (104, 354)	0.053
Plasma renin activity (ng/(mL×h)), median (IQR)	1 (1, 4)	1 (0, 1)	2 (1, 5)	0.053
Epinephrine (pmol/L), median (IQR)	13 (10, 24)	23 (20, 24)	12 (10, 14)	0.190
Norepinephrine (pmol/L), median (IQR)	400 (245, 449)	409 (346, 449)	352 (207, 472)	0.460

Abbreviations: ACEi, Angiotensin‐converting enzyme inhibitor; ARB, angiotensin receptor blocker; BMI, Body mass index; BNP, B‐type natriuretic peptide; eGFR, effective glomerular filtration rate; FeNa, fractional excretion of sodium; HFpEF, heart failure with preserved ejection fraction; IQR, interquartile range; K, Potassium; LVEF, left ventricular ejection fraction; Na, Sodium; NT‐proBNP, N‐terminal pro B‐type natriuretic peptide; NYHA, New York Heart Association; SD, Standard deviation; Wt, Weight.

### 3.1 Response to saline infusion and furosemide administration

**FIGURE 1:**
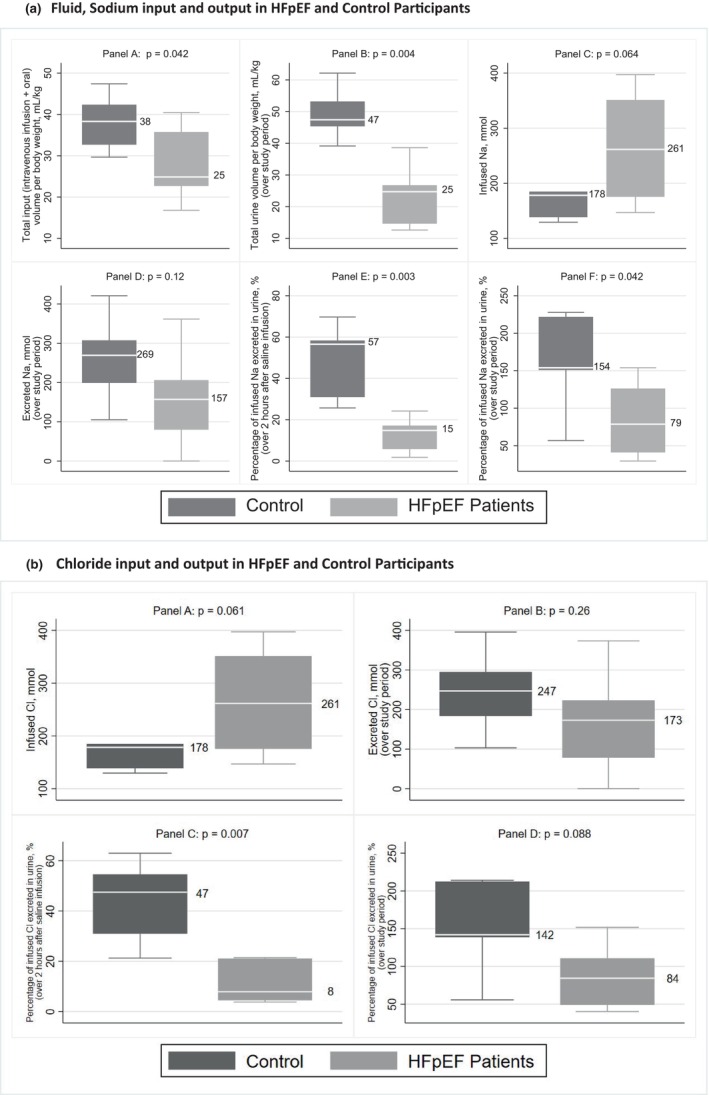
(a). Fluid, Sodium input and output in HFpEF and Control Participants. (b). Chloride input and output in HFpEF and Control Participants. Presented are median (IQR). *p*‐values from Wilcoxon rank‐sum test. HFpEF, Heart failure with preserved ejection fraction; IQR, interquartile range; Cl, chloride.

Saline infusion dose per kilogram body weight was the same in both groups (0.25 mL/kg/min for 60 min), while the total fluid intake (including oral water intake) per kilogram body weight was lower in HFpEF patients (median 25 vs. 38 mL/Kg) (Figure 1, panel a). Patients with HFpEF also had significantly lower urine volumes relative to controls per Kg body weight over the study period (median 25 mL/kg vs 47 mL/kg) (Figure 1, panel b). Even though the amount of infused sodium was higher (median 261 mmol vs 178 mmol) in patients with HFpEF, the amount of sodium excreted over the study period was lower (median 157 mmol vs. 269 mmol) (Figure 1, panel c,d). The percentage of intravenously infused sodium excreted in the urine was also significantly lower in HFpEF patients compared to that in controls (Figure 1, panel e,f). The difference in the percentages of infused sodium excreted between the two groups is shown in Table 2.

**TABLE 2:** Difference between the groups in %age of sodium excreted in urine, and baseline urinary cGMP/plasma BNP ratios, adjusted for baseline variables.

Adjusted for baseline variable	Difference between groups (HFpEF minus control) in % age of infused sodium excreted over study period, mean (95% CI)	Difference between groups (HFpEF minus control) in baseline urinary cGMP/creatinine: Plasma BNP, mean (95% CI)
Unadjusted	−80% (−154, −7)	−3.5 pmol/mg (−7.5, 0.6)
Estimated Glomerular Filtration Rate (eGFR)	−103% (−213, 7)	0.7 pmol/mg (−4.3, 5.8)
Body Mass Index (BMI)	−53% (−151, 46)	−6.5 pmol/mg (−11.0, −1.6)
Age	−90% (−176, −5)	−1.8 pmol/mg (−6.2, 2.5)

Abbreviations: BNP, B‐type natriuretic peptide; CI, Confidence Interval; cGMP, cyclic guanosine monophosphate; HFpEF, Heart failure with preserved ejection fraction.

Plasma BNP, NT‐proBNP, serum aldosterone, and serum osmolality were higher, while FeNa (and FeChloride) were lower in patients with HFpEF as compared to controls at all study time points (Table S1). The “diuretic efficiency” (calculated by ratio of urinary sodium/furosemide dose) was lower in HFpEF patients (mean, 3.0 mmol/mg) compared to that in controls (mean, 7.35 mmol/mg) (*p* = 0.007).

### 3.2 Renal response to endogenous BNP

**FIGURE 2:**
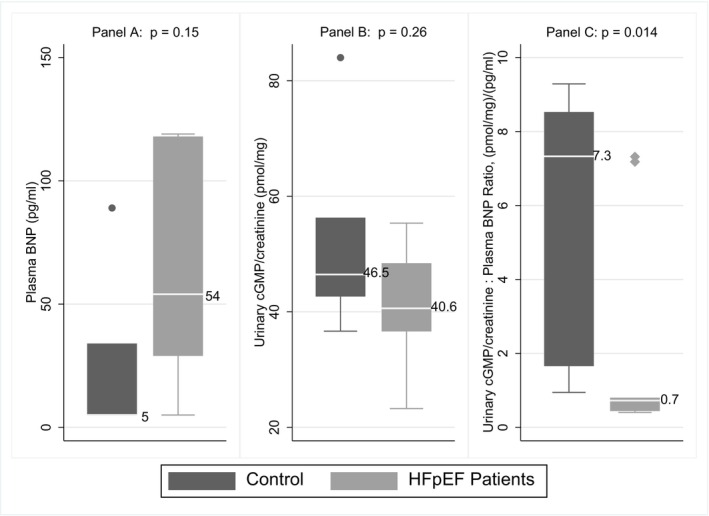
Baseline plasma BNP, urinary cGMP in HFpEF and control participants. Presented are median (IQR) and outliers beyond 1.5 IQRs marked as dots instead of whiskers. *p*‐values from Wilcoxon rank‐sum test. BNP, B‐type natriuretic peptide; cGMP, cyclic guanosine monophosphate; HFpEF, Heart failure with preserved ejection fraction; IQR, Interquartile range.

At baseline, plasma BNP was higher in patients with HFpEF as compared to controls (54 [median IQR 29, 118] vs. 5 [median IQR 5, 34] pg/mL, *p* = 0.15), while the urinary cGMP (normalized for urinary creatinine) was similar between the two groups (median values 40.6 [IQR 36.6, 48.4] vs. 46.5 [IQR 42.6, 56.3] pmol/mg, *p* = 0.26) (Figure 2, panels a, b). The urinary cGMP/ plasma BNP ratio (renal response to BNP) was significantly lower in patients with HFpEF as compared to controls (0.7 [median IQR 0.4, 0.8] vs. 7.3 [median IQR 1.7, 8.5] pmol/mL)/(mg/dL)/(pg/mL), (*p* = 0.014). (Figure 2, panel c). The differences in urinary cGMP/plasma BNP ratio between the two groups (unadjusted and adjusted for baseline eGFR, BMI and age) are shown in Table 2.

From baseline to end of the study, there was a decline in urinary cGMP/plasma BNP ratio in both groups, modestly in patients with HFpEF, from 0.7 [IQR 0.4, 0.8] to 0.4 [IQR 0.1, 1.1] (pmol/mL)/(mg/dL)/(pg/mL), but much more substantially in the control group from 7.3 [IQR 1.7, 8.5] to 0.4 [IQR 0, 1.0] (pmol/mL)/(mg/dL)/(pg/mL) (Table S2).

## 4 DISCUSSION

In this pilot study we demonstrate that compared to healthy controls, patients with HFpEF have reduced urinary sodium excretion and urine output in response to intravenous saline and diuretic challenges, and an attenuated renal response to endogenous BNP.

The biological mechanisms responsible for fluid overload in HFpEF remain uncertain. (Tschope et al., 2017) An abnormal sodium balance in HFpEF patients in response to sodium intake has been postulated, although in contrast to HFrEF, (Nijst et al., 2017) had never been directly demonstrated. (Fang, 2016; Mullens et al., 2017) In contrast to Mckie (McKie et al., 2011) et al who investigated renal sodium handling in patients with preclinical “diastolic dysfunction”, we investigated renal sodium handling in patients with HFpEF. Discordance in the treatment benefits of neurohormonal antagonism in HFpEF versus in HFrEF suggests that despite HFpEF and HFrEF sharing clinical features of volume overload, exercise intolerance, and increased mortality, the pathophysiology of HFpEF may not involve adrenergic or renin‐angiotensin neurohormonal activation as a critical mechanism. (Oghlakian et al., 2011).

We found increased fluid and sodium retention in HFpEF in response to intravenous fluids, which persisted when controlled for baseline eGFR (Table 2), suggesting that the difference in baseline renal function (eGFR) between the groups was not primarily responsible for the difference in renal sodium and fluid handling. Other mechanisms including response to endogenous circulating NPs, and/or increased tubular sodium reabsorption might be at play. Indeed, distal tubular remodeling has been found in HF patients on chronic diuretic therapy. (Rao et al., 2017) We also found lower “diuretic efficiency” in participants with HFpEF; this has been shown to be associated with worse prognosis in acute decompensated HF, however, the significance of this metric has not been validated in compensated HF. (Testani et al., 2014).

We also found an attenuated renal response to endogenous BNP in patients with HFpEF. NPs, including BNP, have natriuretic, vasodilatory, and anti‐fibrotic physiological effects. (Brunner‐La Rocca & Sanders‐van, 2019; Volpe et al., 2016) NP elevation in HF is a counter‐regulatory response to the sodium and volume retentive effects of RAAS activation. cGMP is an intracellular messenger of NPs, and the renal response to NPs can be assessed by measuring urinary cGMP. (Butt et al., 2023; Margulies et al., 1991; Wong et al., 1988) We found that renal responsiveness to plasma BNP, as measured by urinary cGMP/plasma BNP ratio, was significantly *lower* in patients with HFpEF as compared to that in controls. Renal hypo‐responsiveness to BNP could explain, at least in part, the sodium avidity seen in patients with HFpEF. The improvement in symptoms in patients with HFpEF treated with sacubitril/valsartan in the HFpEF trial, PARAGON‐HF, would be consistent with our findings implicating the NP system. (Solomon et al., 2019).

Interestingly, the urinary cGMP/plasma BNP ratio (renal response to BNP) was not lower in HFpEF patients when controlled for baseline eGFR (Table 2). Due to the pilot nature of our study, we cannot confirm whether this was due to chance or whether renal function (eGFR) is the primary determinant of renal responsiveness to plasma BNP in HFpEF.

The benefits of SGLT2i in HFpEF patients suggest a renal contribution from tubular cotransporters to the congestion in HFpEF. Furthermore, positive sodium balance itself leads to endothelial dysfunction, inflammation, and increased vascular and myocardial stiffness, which are common to HFpEF. (Fang, 2016) Indeed, increased sodium has been shown to increase endothelial stiffness and may be implicated in end‐organ fibrosis. (Oberleithner et al., 2007) Thus, it is plausible that increased sodium avidity is the upstream pathophysiological event that leads to a vicious cycle of endothelial dysfunction, inflammation, and myocardial/renal fibrosis in HFpEF.

As a pilot study, our study had several limitations including that we enrolled only a limited number of participants. As expected, the HFpEF patients had a higher co‐morbidity and medication burden compared to control participants who had no history of kidney or cardiac disease, although we did analyze the effects of controlling for baseline variables. A potential role of insulin‐mediated anti‐natriuresis (Brands, 2018) was not assessed. In addition, even though we asked the participants to consume a low sodium diet for 1 week before study, we could not confirm the adherence to this protocol. Additionally, all patients identified as Caucasian, limiting the generalizability of the study. A larger, multicenter study with matched control participants, rigorous assessments of baseline dietary sodium intake, assessments of fractional excretion of endogenous lithium to assess proximal tubular sodium handling (Rao et al., 2024) and proteomic analysis of urinary exosomes would minimize the limitations and expand on the possibilities that this study presents.

In summary, we demonstrate reduced urinary sodium excretion and urinary volumes in response to intravenous saline and furosemide administration, and an attenuated renal response to endogenous BNP in patients with HFpEF. Our findings suggest that an attenuated renal response to endogenous BNP may contribute to impaired natriuresis seen in HFpEF and to consider whether a primary increase in renal sodium avidity could, in addition to other previously characterized mechanisms, contribute as a primary upstream pathophysiological mechanism for volume overload in HFpEF.

## FUNDING INFORMATION

School of Medicine seed grant (no.51900436), and the Divisions of Nephrology and Cardiovascular Medicine, Department of Internal Medicine, University of Utah, Salt Lake City, UT.

## ETHICS STATEMENT

This study was approved by the University of Utah Institutional Review Board (IRB # 00157119) in accordance with the Declaration of Helsinki.

## Supporting information


Data S1.
