# Supplementary material for: Evaluation of renal sodium handling in heart failure with preserved ejection fraction: A pilot study
Source: Physiol Rep. 2024 May 13;12(9):e16033. doi: 10.14814/phy2.16033 (PMC11090885; doi:10.14814/phy2.16033)
Supplement: Supplementary file 1 — Data S1. [file PHY2-12-e16033-s001.docx]

**Supplemental Table 1.**

Serum and Urinary Components at Baseline, Post-Saline, and Post-Furosemide

|  | Baseline | | | 2 hours post-saline infusion | | | 2 hours post-furosemide | | |
| --- | --- | --- | --- | --- | --- | --- | --- | --- | --- |
|  | Control | HFpEF | p-value | Control | HFpEF | p-value | Control | HFpEF | p-value |
| ***Serum Electrolytes*** | | | | | | | | | |
| Na (mmol/L),  median (IQR) | 141  (139, 141) | 136  (134, 138) | 0.030 | 141  (141, 141) | 138  (134, 138) | 0.018 | 142  (141, 142) | 137  (135, 139) | 0.018 |
| Cl (mmol/L),  median (IQR) | 108  (107, 108) | 101  (100, 106) | 0.082 | 109  (109, 110) | 106  (100, 109) | 0.2 | 103  (103, 104) | 104  (100, 109) | 1 |
| K (mmol/L),  median (IQR) | 3.9  (3.9, 4.1) | 4.2  (4.0, 4.6) | 0.140 | 4  (4, 4) | 4  (4, 5) | 0.16 | 3.9  (3.7, 4.0) | 4.2  (3.6, 4.5) | 0.16 |
| Creatinine (mg/dL), median (IQR) | 0.8  (0.8, 0.9) | 1.2  (1.0, 1.5) | 0.009 | 1  (1, 1) | 1  (1, 1) | 0.013 | 0.7  (0.7, 0.9) | 1.1  (1.0, 1.4) | 0.006 |
| *Neurohormones and serum osmolality* | | | | | | | | | |
| Aldosterone (ng/dL), median (IQR) | 6  (5, 6) | 18  (14, 26) | 0.003 | 5  (4, 5) | 18  (9, 27) | 0.004 | 7  (6, 8) | 18  (16, 32) | 0.004 |
| BNP (pg/ml),  median (IQR) | 5  (5, 34) | 54  (29, 118) | 0.150 | 13  (11, 31) | 52  (23, 116) | 0.24 | 15  (5, 31) | 87  (34, 119) | 0.14 |
| NT pro-BNP (pg/ml), median (IQR) | 18  (15, 43) | 197  (104, 354) | 0.053 | 19  (18, 59) | 187  (143, 321) | 0.072 | 30  (24, 57) | 198  (180, 439) | 0.072 |
| Renin activity (ng/(mL*hour)),  median (IQR) | 1  (0, 1) | 2  (1, 5) | 0.053 | 1  (0, 1) | 1  (1, 4) | 0.31 | 1  (1, 1) | 3  (1, 6) | 0.061 |
| Epinephrine (pmol/L), median (IQR) | 23  (20, 24) | 12  (10, 14) | 0.190 | 11  (10, 15) | 18  (11, 30) | 0.19 | 12  (10, 13) | 18  (10, 26) | 0.19 |
| Norepinephrine(pmol/L), median (IQR) | 409  (346, 449) | 352  (207, 472) | 0.460 | 384  (311, 398) | 371  (235, 488) | 1 | 432  (246, 486) | 490  (288, 669) | 0.56 |
| ***Urine measurements*** | | | | | | | | | |
| K (mmol/L),  median (IQR) | 43  (30, 45) | 55  (35, 89) | 0.500 | 11  (11, 20) | 31  (20, 40) | 0.14 | 11  (11, 12) | 14  (12, 20) | 0.2 |
| Creatinine (mg/dL), median (IQR) | 80  (53, 121) | 105  (84, 161) | 0.260 | 17  (10, 21) | 46  (20, 69) | 0.028 | 6  (5, 10) | 10  (7, 12) | 0.28 |
| Cl (mmol/L),  median (IQR) | 80  (72, 107) | 45  (28, 67) | 0.028 | 70  (43, 84) | 47  (29, 55) | 0.32 | 112  (71, 122) | 113  (98, 126) | 0.55 |
| Osmolality (mOsm/kg), median (IQR) | 447  (442, 629) | 634  (474, 734) | 0.260 | 256  (191, 259) | 339  (274, 403) | 0.096 | 269  (262, 286) | 281  (270, 302) | 0.38 |
| Na (mmol/L),  median (IQR) | 77  (76, 99) | 68  (28, 79) | 0.120 | 86  (52, 93) | 53  (20, 71) | 0.46 | 119  (80, 124) | 108  (84, 122) | 0.95 |
| Urine Volume (ml), median (IQR) |  |  |  | 1039  (868, 1253) | 400  (350, 936) | 0.028 | 1992  (1584, 2108) | 1489  (1185, 1910) | 0.17 |
| FeNa (%), median (IQR) | 0.006  (0.004, 0.008) | 0.004  (0.002, 0.008) | 0.26 | 0.017  (0.016, 0.021) | 0.008  (0.006, 0.015) | 0.020 | 0.116  (0.016, 0.124) | 0.082  (0.067, 0.125) | 0.95 |
| FeK (%), median (IQR) | 0.118 (0.070, 0.198) | 0.131 (0.091, 0.186) | 0.74 | 0.238 (0.161, 0.246) | 0.161 (0.104, 0.189) | 0.32 | 0.341 (0.250, 0.370) | 0.390 (0.334, 0.526) | 0.55 |
| FeCl (%), median (IQR) | 0.009 (0.006, 0.010) | 0.005 (0.002, 0.008) | 0.16 | 0.021 (0.018, 0.024) | 0.012 (0.010, 0.012) | 0.006 | 0.150 (0.020, 0.166) | 0.132 (0.097, 0.155) | 0.84 |

Abbreviations: BNP: B-type natriuretic peptide; FeNa: Fractional excretion of sodium; FeK: Fractional excretion of potassium; FeCl: Fractional excretion of chloride; HFpEF: Heart failure preserved ejection fraction; K: Potassium; Na: Sodium; NT-proBNP: N-terminal pro B-type natriuretic peptide

**Supplemental Table 2.**

Urinary cGMP and ucGMP/Plasma BNP Ratios at Baseline and Post-Furosemide

|  | Baseline | | | After saline and furosemide | | |
| --- | --- | --- | --- | --- | --- | --- |
|  | Controls (n=5) | HFpEF (n=9) | p-value | Controls (n=5) | HFpEF (n=9) | p-value |
| Plasma BNP (pg/ml),  median (IQR) | 5 (5, 34) | 54 (29, 118) | 0.15 | 15 (5, 31) | 87 (34, 119) | 0.14 |
| Urinary cGMP (pmol/ml),  median (IQR) | 445 (293, 516) | 469 (384, 500) | 0.64 | 8 (0, 18) | 27 (7, 64) | 0.38 |
| Urinary creatinine (mg/dL), median (IQR) | 80 (53, 121) | 105 (84, 161) | 0.26 | 6 (5, 10) | 10 (7, 12) | 0.28 |
| Urinary cGMP: creatinine ((pmol/ml)/(mg/dL)), median (IQR) | 46.5 (42.6, 56.3) | 40.6 (36.6, 48.4) | 0.26 | 5.3 (0.0, 31.3) | 17.3 (7.0, 63.2) | 0.46 |
| Urinary cGMP: plasma BNP ((pmol/ml)/(pg/ml)), median (IQR) | 48.3 (22.4, 58.6) | 8.7 (4.2, 17.2) | 0.21 | 0.3 (0.0, 1.2) | 0.6 (0.1, 3.7) | 0.29 |
| Corrected* urinary cGMP: BNP ((pmol/ml)/(mg/dl)/(pg/ml)) median (IQR) | 7.3 (1.7, 8.5) | 0.7 (0.4, 0.9) | 0.014 | 0.4 (0.0, 1.0) | 0.4 (0.1, 1.1) | 0.68 |
| Urinary cGMP: plasma NT pro-BNP ((pmol/ml)/(pg/ml)), median (IQR) | 16.3 (10.4, 20.1) | 3.1 (1.1, 4.8) | 0.053 | 0.0 (0.0, 0.6) | 0.2 (0.0, 0.8) | 0.29 |
| Corrected* urinary cGMP: NT pro-BNP ((pmol/ml)/(mg/dl)/(pg/ml)) median (IQR) | 2.0 (2.0, 2.8) | 0.2 (0.1, 0.3) | 0.014 | 0.2 (0.0, 0.2) | 0.1 (0.0, 0.3) | 0.68 |

Abbreviations: BNP: B-type natriuretic peptide; cGMP: Cyclic guanine monophosphate; Cr: Creatinine; HFpEF: Heart failure preserved ejection fraction; ucGMP: Urinary cyclic guanine monophosphate. * Corrected for dilution with urinary creatinine.

**Supplemental Figure; Study Design**


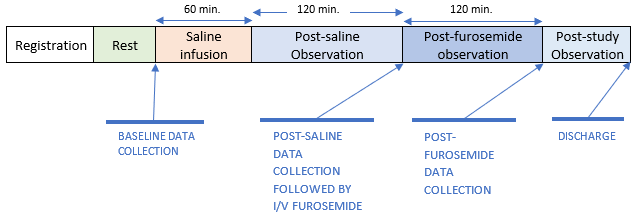


Baseline data collection was followed by saline infusion over one hour, followed by data collection after 120 minutes, then furosemide was administered which was followed by data collection after 120 minutes.

Abbreviations: min.: minutes, i/v: intravenous
